# Gap-filling of ocean color over the tropical Indian Ocean using Monte-Carlo method

**DOI:** 10.1038/s41598-022-22087-2

**Published:** 2022-11-01

**Authors:** Aditi Modi, M. K. Roxy, Subimal Ghosh

**Affiliations:** 1 Centre for Climate Change Research, Indian Institute of Tropical Meteorology, Pune, India; 2 IDP in Climate Studies, Indian Institute of Technology, Bombay, India; 3 Department of Civil Engineering, Indian Institute of Technology, Bombay, India

**Keywords:** Climate-change impacts, Physical oceanography, Carbon cycle, Marine biology, Scientific data

## Abstract

Continuous remote-sensed daily fields of ocean color now span over two decades; however, it still remains a challenge to examine the ocean ecosystem processes, e.g., phenology, at temporal frequencies of less than a month. This is due to the presence of significantly large gaps in satellite data caused by clouds, sun-glint, and hardware failure; thus, making gap-filling a prerequisite. Commonly used techniques of gap-filling are limited to single value imputation, thus ignoring the error estimates. Though convenient for datasets with fewer missing pixels, these techniques introduce potential biases in datasets having a higher percentage of gaps, such as in the tropical Indian Ocean during the summer monsoon, the satellite coverage is reduced up to 40% due to the seasonally varying cloud cover. In this study, we fill the missing values in the tropical Indian Ocean with a set of plausible values (here, 10,000) using the classical Monte-Carlo method and prepare 10,000 gap-filled datasets of ocean color. Using the Monte-Carlo method for gap-filling provides the advantage to estimate the phenological indicators with an uncertainty range, to indicate the likelihood of estimates. Quantification of uncertainty arising due to missing values is critical to address the importance of underlying datasets and hence, motivating future observations.

## Introduction

The tropical Indian Ocean exhibits two annual blooms of phytoplankton with the highest peak occurring during summer (June–September) and a secondary peak during winter (December–February)^1,2^. These blooms are driven by the changes in the physical forcing primarily associated with the southwest (summer) and northeast (winter) monsoon^3–6^. The phytoplankton blooms regulate the food availability for higher trophic levels, making primary production central to both the aquatic food web and the Indian Ocean rim population that is dependent on marine fisheries for their livelihood^7–11^. Recent decades have observed warming of the earth’s climate unequivocally, with the oceans accounting for approximately 93% of this increased energy uptake^12,13^. Amongst the tropical oceans, the Indian Ocean has undergone the largest warming (0.15 °C/decade) in ocean surface^11,14,15^, with projections of a stronger warming (> 1.5 °C) by 2070 and (> 2.5 °C) by 2100 across the CMIP5 models^16,17^. In the low-latitude regions, a warmer ocean surface enhances the ocean stratification thereby reducing the vertical mixing and inhibiting the nutrients (required for photosynthesis) into the sunlit zone of the ocean^18,19^. This limits the marine primary production subsequently impacting the biodiversity of the ocean^11,20,21^. Any further warming is therefore expected to affect both the mean biomass and the timings of seasonal phytoplankton blooms in tropical ecosystems^22,23^. The timing of phytoplankton blooms, known as phenology, directly affects the larval spawning and survival. For example, the onset of local phytoplankton bloom marks the hatching of pink shrimps in North Atlantic^24,25^. Any change in the bloom initiation timings are therefore likely to proliferate across the higher trophic levels—popularly known as the match-mismatch hypothesis^26^. Phenology has been argued to be one of the most sensitive biological indicators to detecting changes in the marine ecosystem by the Intergovernmental Panel on Climate Change^27^. With the increasing diversity and resolution of our observations, it is imperative to examine the response of ecological indicators of the marine ecosystem to the rapidly warming tropical oceans, and ocean color is currently the only window to understand the impact of the changing climate on ocean biology; therefore classified as an essential climate variable^28^.

To date, cost-effective, routine spatio-temporal observations of phytoplankton biomass have been remarkably possible only through the satellite ocean color sensors making it vital in operational forecasting, oceanographic research and numerical modelling of climate^29,30^. Remote-sensed ocean color provides measurements of chlorophyll—the phytoplankton pigment that undergoes photosynthesis and is a proxy to marine phytoplankton^31^. The ocean color fields as measured by SeaWiFS (1997–2010), MODIS (2002–), MERIS (2002–2012), VIIRS (2011–) sensors span different time periods with very limited overlapping between the two missions. In view of this, a recent Ocean-Color Climate Change Initiative (OC-CCI) by the European Space Agency (ESA) provides a high-quality, long-term chlorophyll dataset at a very high resolution (~ 4 km), achieved by blending ocean color observations from multiple satellite missions and applying quality corrections^32,33^—thus bringing the current global records of chlorophyll to more than two decades (22 years). The availability of OC-CCI chlorophyll data has resulted in a consistent rise in the assessment of trends in the marine ecosystem; therefore expanding our ability to detect the signature of human-induced climate change on the marine ecosystem^33,34^. Previous studies suggest a decline in the marine primary production throughout the tropical oceans, particularly the open oceans and have warned of detrimental impacts on the marine food web in response to future ocean warming^35–38^. The western Indian Ocean, which is the most biologically productive region of the Indian Ocean, has already undergone a significant decline of 20–30% in the surface phytoplankton distribution during 1998–2013, as shown by both satellite observations and the CMIP5 Earth System Models^38^. However, the phenology of the seasonal phytoplankton blooms is still overlooked in the Indian Ocean, majorly due to the lack of gap-free observations at a higher temporal frequency.

Satellites have been instrumental in advancing our knowledge of the changing biophysical interactions under the recent climate change scenario. Besides global coverage, data obtained from satellites are high in both sampling frequency and spatial resolution required to assess trends and interannual variability of phytoplankton phenology^39^. However, satellite observations are subject to gaps—both frequent and persistent—caused by several factors such as sun-glint, atmospheric aerosols, cloud cover, sensor saturation, hardware issues^40–42^. Missing data tends to reduce the statistical power of a dataset along with affecting the accuracy of the estimating parameters^43^. The merging of chlorophyll by OC-CCI has led to a reduction in missing values^40^ as a result of the overlap in the spatial coverage of satellite sensors. However, this does not solve the critical issue of gaps in satellite data occurring due to the presence of clouds^44,45^.

**Figure 1:**
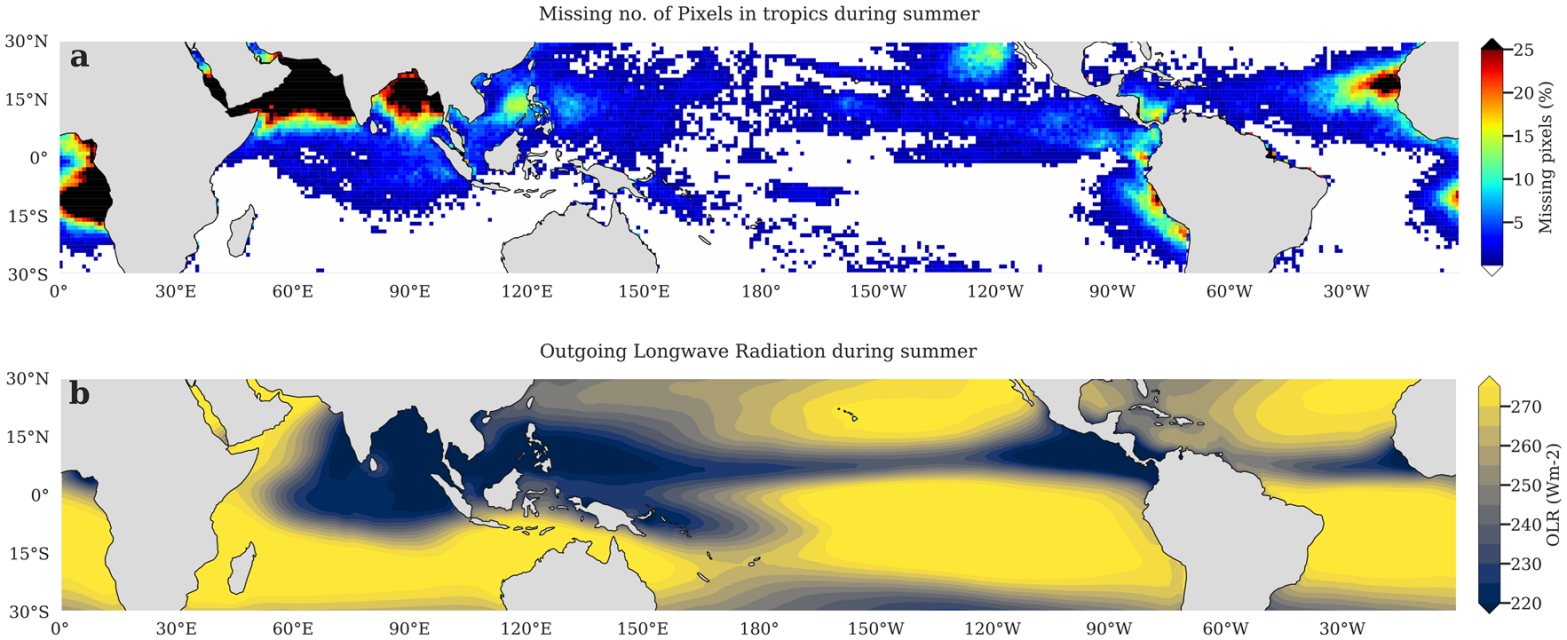
Missing values in ESA chlorophyll and mean OLR in the tropics during boreal summer. (**a**) Number of missing pixels (in percentage) in 8-day composites of ESA OC-CCI chlorophyll data from 1998–2019 over the tropical oceans during boreal summer (June–September). Gap-free pixels are indicated in white. The pixels having more than 25% of missing values are shown in black; and (**b**) Climatological map of OLR (in W/m^2^) during summer (June–September) for the tropical oceans for the period 1998–2019. The regions in dark blue are associated with a weaker convection and those represented in yellow represent strong convection. This figure is created using Python 3.9.10 software (https://docs.python.org/release/3.9.10/).

Over the tropical Indian Ocean during the summer monsoon, the gaps in ocean color data can be as high as 40% (Fig. 1a). Enhanced convective activity owing to moisture-laden monsoonal cross-equatorial flow during June–September leads to formation of persistent cloud cover over the Indian Ocean north of 10S^46,47^. This results in a considerable reduction in outgoing longwave radiation (OLR) by up to 100 W/m^2^ from the annual mean (Fig. 1b), hence preventing the satellites from observing the ocean^48^. This poses a major challenge in the usage of satellite data as it is during the summer monsoon season that the highest productivity, driven by enhanced vertical mixing and offshore wind-driven upwelling, is experienced in the north Indian Ocean^3,6^. Furthermore, it is important to note that contrary to the Indian Ocean, satellites have good data coverage over the Pacific and Atlantic (Supplementary Fig. S1). This may be one of the factors leading to the fact that the Indian Ocean is least understood of all the tropical basins^38^. Some of the highly productive regions in the tropical oceans include eastern Pacific and eastern Atlantic. However, due to low cloudiness (reflected in the OLR, Fig. 1b), both the regions have a good satellite coverage^49^. Hence, in studies that consider the global domain, this difference of cloud cover in the different basins can bias the regional estimates of both the phytoplankton distribution and phenology^44,50^ and needs to be considered. The potential biases introduced by intermittent data in assessment of ecological trends and variability has already been demonstrated in the scientific literature. Errors of typically 15 and 30 days in the bloom peak and initiation timings respectively and high uncertainty (> 2 weeks) in the duration of the seasonal phytoplankton bloom have been estimated when dealing with incomplete time series^44,51^; making gap-filling a prerequisite for detecting the phenological (or, ecological) indices of the ocean ecosystem using the existing observations.

Most common gap-filling techniques of ocean color data involve spatial or temporal interpolation^51–53^; filtering^54,55^; and substitution by mean, median, minimum^56^. Conventionally, interpolation has been the most widely used tool in scientific literature to deal with data-gaps. It involves extending the areal coverage of the data by utilizing the information of the neighboring observations. However, if not performed cautiously, excessive smoothing (or, interpolation) can disrupt data quality by blending the sub-grid to grid scale features, leading to under- or over-estimation of the chlorophyll concentrations; thereby making the data unreliable for extracting information of the important local biophysical processes. This is because linear interpolation is based on the assumption that the missing pixel has a linear relationship with the surrounding pixels which is far from reality, as phytoplankton blooms are known to occur in patches and their concentration varies dramatically from the coastal to the open ocean waters. Hence, beyond a certain neighboring grid, interpolation of ocean color data becomes invalid^57^. Additionally, averaging the data to a coarser temporal resolution of the order of monthly scale might reduce the frequency of gaps but the reduced time scale will leave the data inadequate for estimation of phenological indicators. Similarly, substituting the missing value with the sample average seems convenient as a method of gap-filling, but the phenological algorithms to estimate the interannual variability and trends, when applied to the reconstructed data, leads to flawed outcomes. Moreover, since the ocean color satellite data is highly positively skewed (skewness value of log (chlorophyll) = 1.51, Fig. S2), using the data-mean to fill the missing values would be inappropriate for a skewed distribution.

There are a few advanced methods presented in the literature for gap-filling such as neural networks, empirical orthogonal functions (DINEOF); however they are limited to single value imputation overlooking the uncertainty of estimates around the true value—an important aspect of gap-filling^40,52,58^. Most of the studies have utilized a small sample size (< 10 years) of satellite data and has been limited to reconstructing a gap-free climatology, which does not allow for examining the interannual variability and trends. An attempt to prepare a gap-free climatology of chlorophyll for the tropical Indian Ocean employing 7 years of SeaWiFS data has been carried out by Levy et al.^54,55^. It was the first attempt using remote-sensed ocean chlorophyll to produce a gap-filled dataset over the Indian Ocean. However, their methodology is based on filtering techniques, essentially relying on the information of surrounding spatio-temporal values for estimating the missing value and limited to single value imputation. Even with the recent technological advancement and computational resources available, the current set of CMIP6 model outputs of chlorophyll are limited to a monthly scale. Hence, this problem of intermittent datasets needs addressal with the existing satellite ocean color data, which drives the purpose of this study.

The choice of the method becomes increasingly important as the amount of missing data increases such as in the case of the tropical Indian Ocean. More importantly, to draw useful inferences in the phenological indices estimated from these gap-filled datasets, it becomes crucial to address the uncertainty of the estimates. With the single imputation methods used to fill the missing data, no information of the uncertainty associated with the analyzed parameters can be determined. We believe that this issue can be addressed by applying computational statistical tools of moderate complexity. Hence, in this study, we propose a methodology of gap-filling which enables us to quantify the phenological indices along with uncertainty measures. We achieve this by performing a large number of simulations using the classical Monte-Carlo method in addition to a strict optimal local averaging to fill the gaps in ocean color data. The Monte-Carlo procedure allows us to impute the missing pixel with a set of pseudo-random values in place of a single value as done with the other techniques of gap-filling. This approach will generate ensembles of reconstructed datasets rather than just producing a single gap-filled data. This provides the additional advantage of using Monte-Carlo approach, as we will have a range of possible values in the derived phenological parameters. The proposed methodology is explained in the “Methods” section and schematically illustrated in Fig. 3.

The primary emphasis of this study is to acknowledge the importance of determining uncertainty in the estimated parameters derived using these gap-filled datasets which is a significant advantage of using this method, otherwise not possible with the conventional gap-filling approaches. Though focused on marine ecosystems, this methodology can be extended to using other variables of the climate system. We hope that this work contributes towards improving the use of existing datasets to extract reliable information of biophysical processes of the marine ecosystem.

## Data and methods

Daily synoptic fields of remotely-sensed chlorophyll concentrations (in mg/m^3^) at a 4-km spatial resolution were obtained for the period 1998–2019 from the European Space Agency Ocean Color-Climate Change Initiative (OC-CCI) version 4.2 (https://climate.esa.int/en/projects/ocean-colour/). The chlorophyll-*a* time series (chl-*a*) obtained from OC-CCI is a multi-mission product aimed to provide a global long-term dataset to support trend studies in the marine ecosystem, otherwise impossible from single mission products—due to lack of continuity and homogeneity—and is derived by merging data from the SeaWiFS, MODIS, MERIS, and VIIRS sensors^32^. The Level-3 data from different sensors were band-shifted to SeaWiFS wavebands and bias-corrected for the signal-to-noise ratio, thus resulting in a climate-quality dataset^59,60^. Though the satellite ocean color measurements started in 1978 with the CZCS mission, it couldn’t be merged with other sensors owing to its limited spatial coverage and the difference in spectral bands.

Apart from chlorophyll, gridded daily OLR fields provided by NOAA at one-degree resolution are utilized in the study. Moreover, a gap-filled 7-year SeaWiFS climatology (for 1998–2006) reconstructed by Levy et al. (mentioned hereafter as Levy) and is available online (http://www.nio.org).

Bloom initiation and peak are predicted using the threshold method^61^. The bloom initiation is declared when summer chlorophyll exceeds a threshold of 5% above the annual median value. And bloom peak is defined as the maximum chlorophyll value during summer months.

### Gap-filling algorithm

Before applying the gap-filling algorithm, 8-day composites are prepared from the chlorophyll daily time series and then the chlorophyll data is re-gridded from 4 km × 4 km resolution to 1° × 1° using conservative binning. This reduces some gaps in the chlorophyll data and the resultant temporal and spatial resolution is sufficient for applying statistical algorithms for evaluating phenological indicators. Then the gap-filling algorithm is applied to this data in a two-step procedure (Fig. 3) as discussed below.

### Step I: Optimal Linear Interpolation

The first step involves filling in the gaps using an optimal linear interpolation scheme applied along the three dimensions sequentially in the order of longitude, latitude and time^51^. The choice of the sequence of latitude, longitude and time used for interpolation made in the study is based on the underlying physical features observed in case of the phytoplankton blooms. The zonal variation in phytoplankton biomass is comparatively lesser than the meridional variation^6^, hence interpolation is first done longitude-wise and then latitude-wise. For our study, we are using weekly chlorophyll values and the temporal variations in phytoplankton biomass are high at weekly timescales^18,62^. Hence, time is used last for the interpolation. The root mean square error (RMSE) values computed for interpolation in each dimension also confirm to the precedence order used (Supplementary Table 1). Under the scheme, a missing pixel is filled by substituting it with the arithmetic mean of the two neighboring grid values, each weighing equally. If the surrounding point used in averaging is invalid (a land point) or missing (in the ocean), it is assigned a zero weight. If both the points surrounding the missing pixel are found to be invalid along all the three dimensions, then the gap is left unfilled.

### Step II: Monte-Carlo Filling

To fill the remaining gaps, we use Monte-Carlo method in the second step of the gap-filling algorithm. Monte-Carlo involves filling the missing pixel with a set of plausible values instead of a single value and utilizes inferential statistics to provide a probabilistic solution to the problem of interest. In Monte-Carlo, pseudo-random values are drawn based on the probability distribution of data^43,63,64^. Monte-Carlo method is based on the assumption that the true value of the missing pixel lies within the probabilistic distribution of the large population. It involves repeated sampling such that the number of simulations (N) are enough to estimate the probability distribution correctly^65^. To identify the best-fit probability density function (PDF) of the chlorophyll fields for the Indian Ocean at each grid, the parametric distributions (here, normal, lognormal and gamma) are applied. The Kolmogorov–Smirnov (K–S) test confirms how well the assumed distributions fits the data^63^. Using the K–S test, the best-fit curve is obtained based on the largest p-value. If the K–S test suggests a poor-fitting for all the applied parametric distributions (p-value < 0.05) at a grid, then a Kernel density Estimate (KDE) is used to estimate the PDF.

KDE is a non-parametric approach of curve-fitting to estimate the best-fit PDF of the variable. The major advantage of choosing KDE over other non-parametric methods is that it is independent of the bin size and the starting bin and it produces a smooth estimate of the PDF, thus giving a better representation of multimodality^66^. The kernel used in the KDE model is Gaussian and the most critical parameter, bandwidth of the kernel is determined using the cross-validation method. Once the distribution which best fits the data for that grid is identified, we generate 10,000 instances using the identified PDF. It is to be noted that this process of identifying the best-fit PDF is repeated for each grid point and the supplementary Fig. S3 represents the PDF used for each grid. Most of the grids in the North Indian Ocean are fitted with a KDE (Fig. S3). This KDE curve-fitting for most of the grids in our case gives us the advantage that no assumption is made about the data, which makes the Monte-Carlo gap-filling more robust. However, as KDE has a limitation of the bandwidth range, hence we are using the parametric distributions wherever it better fits the data.

Once the input probability distribution of the Monte-Carlo model is identified, the distribution-specific parameters (shape, scale and location) are estimated at each grid using the identified PDF for that grid. These parameters of the assumed probability distribution are then fed to the Monte-Carlo model and the gaps are filled by generating N pseudo-random values using a random number generator. Here, we have performed a significantly large number of Monte-Carlo (N = 10,000) computations. All of these ensembles when used for any subsequent analysis, will lead to a range of estimates allowing the estimation of uncertainty in derived measures such as phenology.

## Results

**Figure 2:**
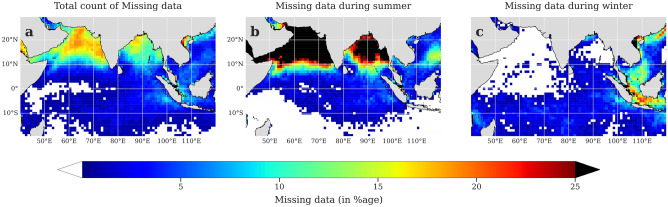
Annual and seasonal maps showing the count of missing pixels (in percentage) in the ESA merged chlorophyll data over the tropical Indian Ocean. Number of missing pixels (in percentage) in the 8-day composites of 22 years of ESA OC-CCI chlorophyll data from 1998–2019 in the Indian Ocean during the months (**a**) January–December; (**b**) June–September; (**c**) December–February. The seasonal maps of (**b**) and (**c**) indicate the seasonal contribution to the total number of observed pixels in (**a**). Gap-free pixels are indicated in white. The pixels exceeding 25% of missing values are marked as black. This figure is created using Python 3.9.10 software (https://docs.python.org/release/3.9.10/).

Missing pixels up to 25% are reflected over the tropical Indian Ocean in the multi-sensor merged chlorophyll dataset (Fig. 2a). These missing pixels are dominant in the summer (Fig. 1a) due to the presence of thick cloud cover indicated by the low OLR over the tropical Indian Ocean (Fig. 1b) which reduces considerably by 100 W/m^2^ with respect to the annual mean. Moreover, the strong convective activity over the Arabian Sea and the Bay of Bengal leads to a strong disproportionality in the percentage of missing data in the two hemispheres (Fig. 2b). Hence, the majority of missing values are present over the north Indian Ocean. Whereas during the winter months, absence of strong convective activity improves the satellite retrieval^3^, leaving no significant gaps in the data over the north Indian Ocean (Fig. 2c).

**Figure 3:**
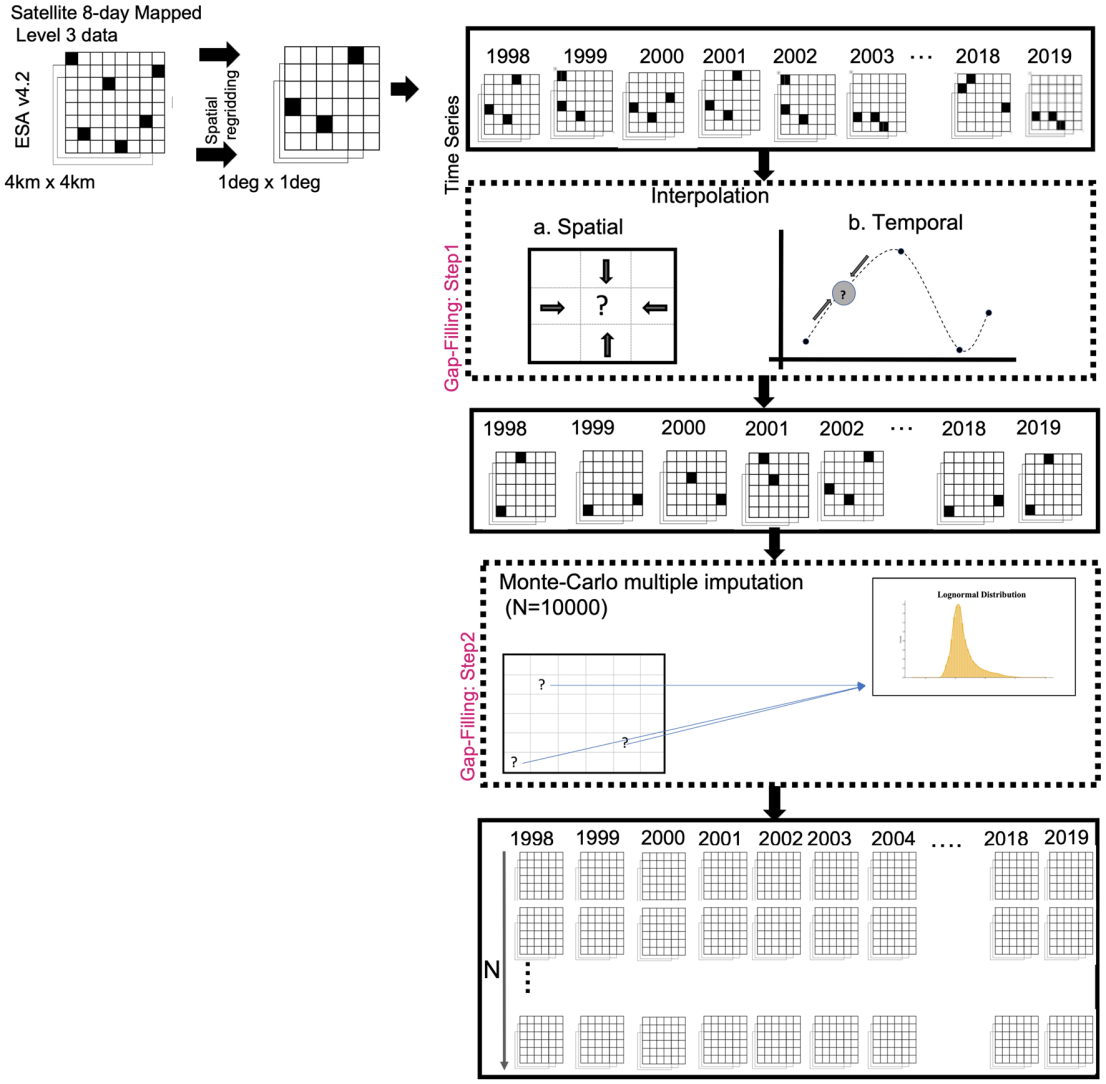
Schematic of the algorithm to fill the gaps of missing chlorophyll concentrations. The daily chlorophyll fields are available at a spatial scale of 4 km × 4 km for the period 1998–2019 by ESA OC-CCI. 8-day composites are prepared from the daily fields and re-gridded to 1 degree to reduce gaps. Then the gaps are filled in two steps: (**a**) Linear Interpolation; (**b**) Monte-Carlo Multiple Imputation. The arrows depict the sequence of the algorithm (refer to “Methods” section for details). Only pixels with missing data are reconstructed. The schematic is adapted from Racault et al.^51^.

**Figure 4:**
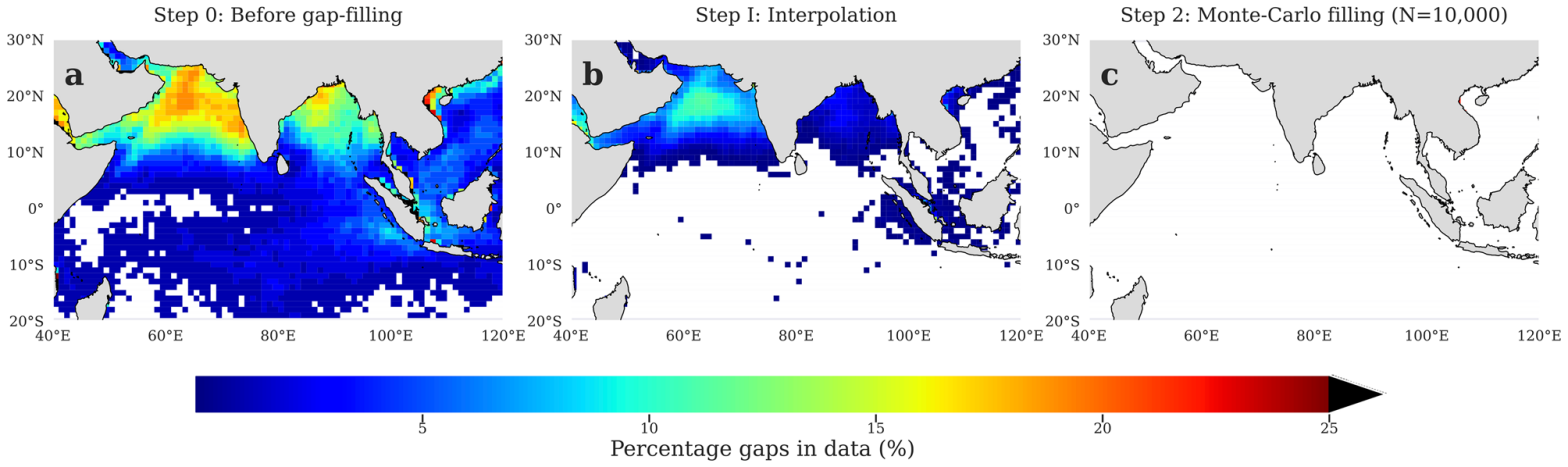
Spatial maps of missing pixels (in percentage) before and after applying the gap-filled methodology. Spatial maps showing missing number of pixels (in percentage) in the 8-day composites of chlorophyll during 1998–2019 in the (**a**) original chlorophyll data from ESA OC-CCI, (**b**) after the first step of interpolation, and (**c**) after the final step of Monte-Carlo multiple imputation. The regions in white indicate gap-free pixels. This figure is created using Python 3.9.10 software (https://docs.python.org/release/3.9.10/).

To reduce these gaps in the basin, we apply our gap-filling algorithm—a two-step procedure. For detailed steps on the methodology, refer to the “Methods” section (Fig. 3). The first step of the algorithm performs optimal linear averaging. This step of the algorithm fills the data-gaps, reducing them by 10% roughly, bringing the data gaps down from 25% (Fig. 4a) to less than 15% in the Arabian Sea and less than 5% in the Bay of Bengal (Fig. 4b). This interpolation step is sufficient to handle the missing pixels of data in the tropical Indian Ocean south of equator as all the gaps are completely filled in the first step. Thereafter, the second step of gap-filling is applied to fill the remaining data-gaps (~ 15%). In this step, Monte-Carlo computations are performed to impute 10,000 ensembles of chlorophyll fields. The filling of gaps with multiple values addresses the problem of wide-gaps present in the data (Fig. 4c).

**Figure 5:**
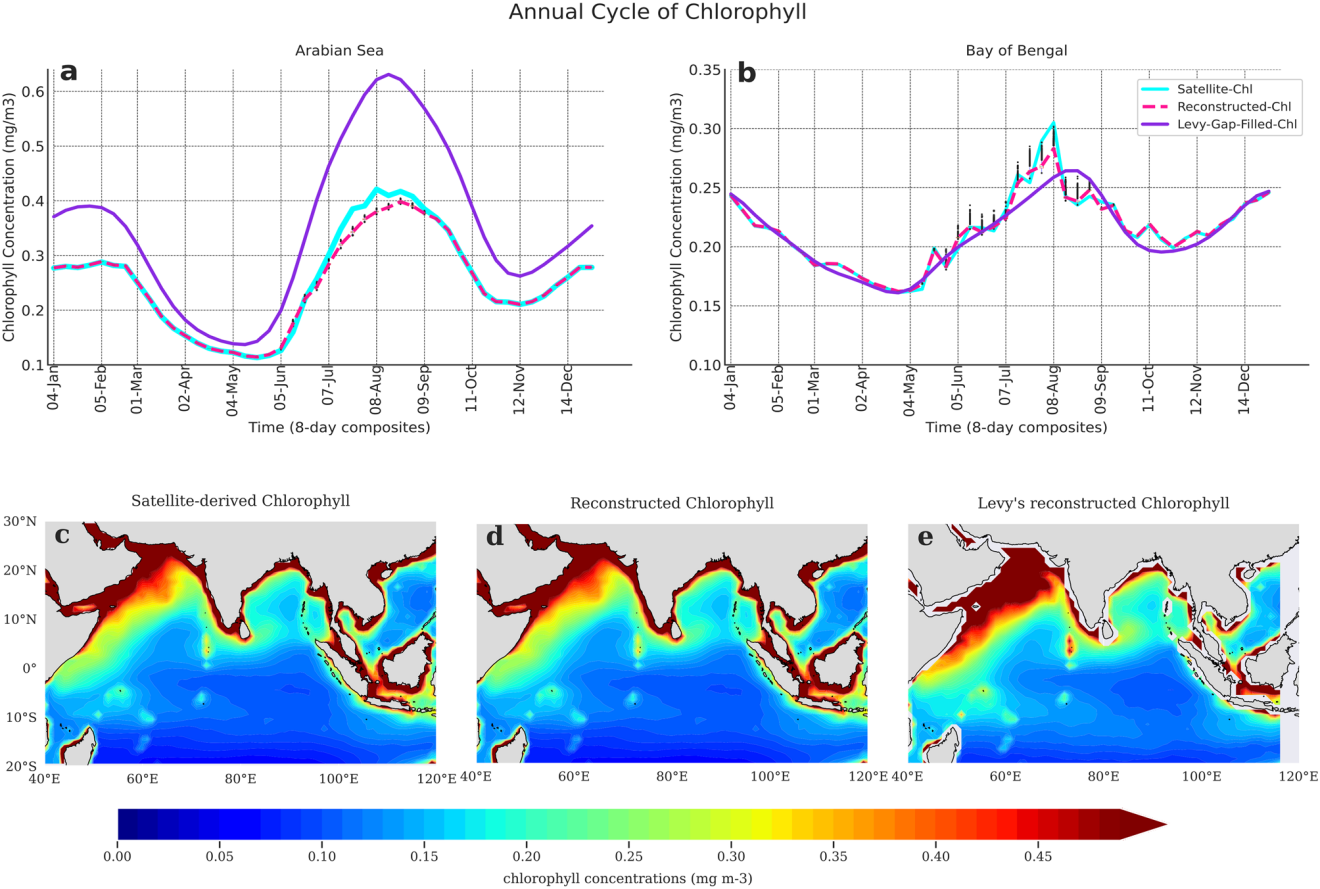
Annual cycle of chlorophyll and its spatial distribution in the Arabian Sea and Bay of Bengal for the original and gap-filled datasets. Climatological annual cycle of reconstructed chlorophyll (8-day composites) for the period 1998–2019 in the (**a**) Arabian Sea [60°E–70°E, 8°N–16°N], and (**b**) Bay of Bengal [85°E–95°E, 8°N–16°N]. Light Blue line indicates ESA v4.2 satellite chlorophyll (original data); pink line represents the mean of gap-filled chlorophyll datasets (reconstructed data); and violet line represents the climatology of the gap-filled annual cycle reconstructed by Levy. The boxplot overlaid on the time series represents the range of values between the 25th and the 75th percentile. The black dots represents the outliers. Spatial distribution of chlorophyll concentration (in mg/m^3^) in the tropical Indian Ocean for the period 1998–2019 in (**c**) satellite chlorophyll, (**d**) reconstructed chlorophyll using our proposed methodology, and (**e**) Levy’s reconstructed dataset. This figure is created using Python 3.9.10 software (https://docs.python.org/release/3.9.10/).

We confirm whether the gap-filling method induces any spurious artifacts in the mean spatial and temporal features in the reconstructed datasets by showing the annual climatological cycle of the ensemble datasets (Fig. 5). The mean of the ensembles (indicated in pink) coincide with the original annual cycle (indicated in light blue) both in amplitude and phase—both for the Arabian Sea and Bay of Bengal^67,68^ (Fig. 5a,b). The uncertainty bands (represented by box and whiskers) provides useful information about the variation of the predicted values by Monte-Carlo for gap-filling. The boxplots clearly shows that 75th percentile of the values lie within a very narrow range with few outliers. This uncertainty band is narrow due to the fact that the data is averaged over a region, but it might be higher for an individual grid. Albeit higher, the uncertainty band is indispensable to the estimated parameters from the gap-filled datasets. Needless to mention, this uncertainty accounts for all the variability—ranging from local to interannual—exhibited by the bloom events. Further examination of the reconstructed chlorophyll ensemble mean is seen to exhibit a similarity in its spatial distribution to the original chlorophyll concentrations (Fig. 5c,d).

In Levy's reconstructed climatology (Fig. 5a, shown in violet), a higher amplitude of chlorophyll annual cycle is seen in the Arabian Sea. Also, very high chlorophyll concentrations in the open ocean region of Arabian Sea and Bay of Bengal are seen in Levy’s reconstructed spatial climatology (Fig. 5e). This might be due to the fact that Levy’s climatology is prepared using the data for a shorter duration (7 years), significantly less than the period of 22 years, as utilized in our study. However, to confirm if this factor can be attributed to the overestimation of the amplitude of the annual cycle in the Arabian sea region, we compared the original satellite dataset and our reconstructed dataset for the same time period as Levy’s, i.e., 1998–2005 (Fig. S4). An overestimation of chlorophyll concentrations in the spatial climatological patterns (Fig. S4a,b) and the amplitude of annual cycle is clearly seen in Levy’s reconstructed dataset (Fig. S4c–e), suggesting potential biases in Levy’s reconstructed climatology. Most importantly, the peak of summer and winter blooms coincide in the original and reconstructed climatology, but a delay of 15 days to 1 month is observed in Levy’s annual cycle (Fig. 5b). However, it cannot be substantially stated whether the smoothing applied or the small sample size is the reason behind the observed shift in Levy’s reconstructed data. It is also possible that the original dataset used by Levy might be subject to biases, all of which needs to be accounted for. Nevertheless, it is quite evident from the above comparison that the observed shifts in annual cycle might lead to erroneous computation of ecological indicators.

The above analysis helps us validate that the Monte-Carlo gap-filling does not lead to variations in the spatial and temporal characteristics of phytoplankton distribution in the reconstructed datasets. Further validation of the reconstructed dataset is done with the available Teledyne/Webb APEX—Argo floats^38,69^ for the Indian Ocean. The satellite and the reconstructed data are averaged over a region within the trajectories of the Argo floats (66–68°E, 8–12°N, within 50 km of the Argo location) and compared for the period during which in-situ data is available (year 2010). Even though the satellite data is averaged closely following the Argo trajectory, the Argo data is available for point locations, hence a one-to-one comparison between the two is not possible. It should also be taken into account that while the satellite data measures chlorophyll integrated over the upper part of the photic zone (which may extend up to a few meters in clear waters), the in-situ Argo data used for comparison is at about 6 m depth. Regardless of these limitations, the annual cycle of chlorophyll is well represented in both the satellite and the reconstructed time series and matches with the Argo time series. Along with, both the time series shows a high correlation (*r* = 0.80) with the Argo data, statistically significant at 95% confidence level (Fig. S5). Moreover, the annual mean computed from the Argo dataset (horizontal dashed yellow line, Fig. S5) for the period April–December 2010 is comparable to the satellite mean and reconstructed data mean (horizontal dashed cyan and pink line, Fig. S5). Furthermore, the satellite and the reconstructed datasets shows a very high correlation of 0.995, thus validating the fact that the Monte-Carlo filling does not induce spurious changes in the mean characteristics of phytoplankton biomass distribution.

**Figure 6:**
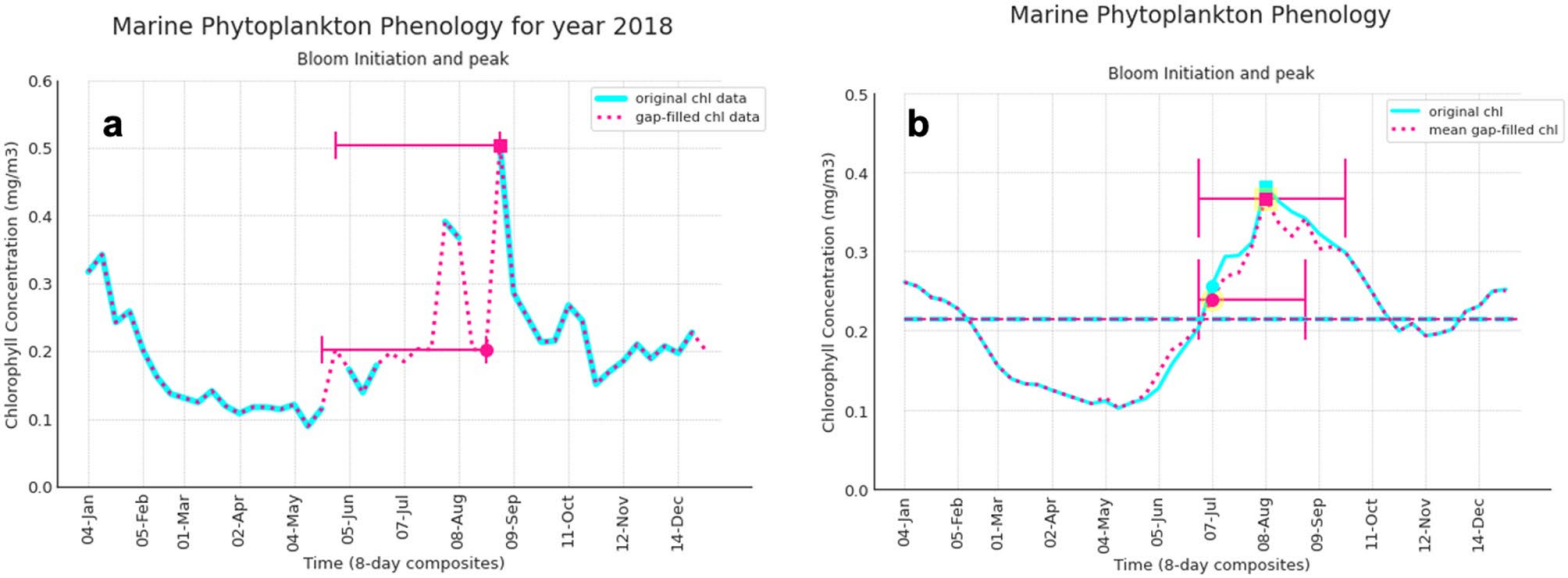
Phenological Indicators derived for the annual cycle of chlorophyll in the Arabian Sea. Bloom initiation and bloom peak estimated for the 8-day composites of the reconstructed datasets showing (**a**) for the year 2018, (**b**) chlorophyll climatology during 1998–2019. The phenological indices shown here are computed for a grid location in the central Arabian Sea [64°E, 11°N]. Light Blue line indicates ESA v4.2 satellite chlorophyll (original data); dotted pink line represents the mean of gap-filled chlorophyll datasets (reconstructed data). The bloom initiation is indicated by solid circles and bloom peak by solid squares in same color as the data. The uncertainty in bloom initiation and peak timings as derived from our gap-filled datasets are represented by a horizontal solid line (pink). The horizontal dashed lines in (**b**) represents the annual median value of both the datasets. This figure is created using Python 3.9.10 software (https://docs.python.org/release/3.9.10/).

The timings of phytoplankton blooms determine the food availability for higher trophic levels in the marine ecosystem. Computing the phenological indices from the time series becomes particularly challenging when data is subject to large gaps. This is where the usage of Monte-Carlo serves its purpose as it enables us to extract the phenological indices along with uncertainty estimates. As a demonstration, we have computed two phenological indicators, bloom initiation and peak, for a recent year in central Arabian Sea which is one of the highest productivity regions in the Indian Ocean. Bloom initiation for the year 2018 is likely to occur between 20th May–24th August (pink horizontal solid line, Fig. 6a) given by the gap-filled ensembles with a mean initiation date as 24th August (pink solid circle) and the bloom peak is likely to occur between 28th May–1st September 2018. This range of bloom indices as given by these ensembles is the uncertainty quantification of the bloom timings (pink horizontal solid line). A high uncertainty is seen here in the initiation and peak which is evident as the data is missing for a longer period during June–September 2018 (light blue line, Fig. 6a). If on the other hand, we compute these indices for a gap-filled data prepared only by single imputation, we fail to extract information of errors arising due to missing values, therefore, missing the estimated uncertainty. Hence, uncertainty quantification is critical especially when gaps are present in the data.

It should be noted that in Fig. 6a the phenological indices cannot be estimated on the original satellite data due to the presence of missing values. Hence, in order to facilitate a comparison between the phenological indices estimated from the reconstructed and original satellite dataset, we prepare climatology of both the datasets to compute the bloom indices. Bloom initiation is likely to occur within 1st–15th July in the gap-filled ensembles (pink solid circle, Fig. 6b) with the mean initiation date as 7th July. The mean bloom initiation time of the reconstructed datasets coincides with the original dataset (light blue, Fig. 6b). Similarly, the bloom peak is likely to occur during 8th–22nd August having a uncertainty band of 2 weeks (pink solid square, Fig. 6b).

## Discussion

Our study is the first to examine the gaps in remotely-sensed ocean color observations in the tropical Indian Ocean and propose a methodology which enables to quantify uncertainty along with the phenological indices when estimated using satellite ocean color observations with missing values. Previous gap-filling techniques applied to ocean color datasets have been limited to single-value imputation which do not provide any information about the uncertainty in the estimated phenological parameters. This uncertainty quantification is overlooked, if conventional gap-filling approaches are adopted and needs to be highlighted which drives the objective of using Monte-Carlo for gap-filling. Using Monte-Carlo method, we fill the missing pixel with multiple pseudo-random values. The outcome of this approach results in multiple gap-free datasets to determine phenological indices with uncertainty values.

We perform an optimal linear interpolation first and it brings down the gaps in the data from 25 to 15% in the North Indian Ocean while making the tropical Indian Ocean south of the equator completely gap-free. Interpolation performed beyond the optimal average^70^ range tends to blend the meso-scale to large-scale features of the data leading to under- or over-estimation of the chlorophyll concentrations. Hence, we have restricted interpolation to only one surrounding grid to ensure optimal local averaging. This still leaves us with gaps in data as high as 15% to which the Monte-Carlo method is applied. Application of Monte-Carlo brings down the percentage of gaps to zero thus making the ocean color dataset gap-free. Using the methodology, we generate 10,000 ensembles of gap-free ocean surface chlorophyll data for the period 1998–2019. All of these ensembles when analyzed, provide a range of estimates accounting for the degree of bias associated with estimating a missing value^58^.

A validation of these reconstructed datasets is done with the satellite data and the available in-situ bio-Argo observations, which shows that the Monte-Carlo filling does not change the spatial and temporal characteristics of phytoplankton biomass. Moreover, generating multiple ensembles for filling missing values also addresses the most critical issue of uncertainty quantification associated with missing data which has been demonstrated in this study by computing the annual timings of bloom initiation and peak (Fig. 6). Since Levy’s climatological dataset is prepared from 7 years of satellite data and with a single satellite sensor, we do not claim that our methodology is generating a better dataset than Levy’s as our emphasis is placed on the most crucial subject which is the quantification of uncertainty in the estimated parameters derived from these gap-filled datasets. Hence, we do not compare the two datasets for absolute values but with the intention to highlight the difference in the outcomes of the two techniques.

Missing data are ubiquitous in remotely-sensed ocean color observations. The seasonally varying cloud cover of the southwest summer monsoon is one of the major reasons for the observed high percentage of missing values over the tropical Indian Ocean. This is also the time when highest productivity is experienced in the northern Indian Ocean. The impact of missing data on statistical inference is potentially significant and are therefore prone to biased estimates^71^; but filling the data-gaps is equally challenging. This paper presents a method to fill gaps in remote sensed data by using sophisticated statistical tools of moderate complexity. The need for these tools is unavoidable as the wider the gaps, the more uncertain are first and second order statistics of the examined time series^70^. Since the chlorophyll observations are global in scope, we expect that this methodology is applicable to the other ocean basins and should not lead to spurious data filling. Though limited to ocean color in the current study, this method can be extended to preparing gap-free datasets of other variables of the earth system. However, the data first needs to be tested for the underlying probability distribution as the power of the proposed statistical algorithm depends on the appropriateness of the assumed underlying distribution. For a more time-specific gap-filling, additional use of the Markov Chains should be made in this method also known as the Markov chain Monte Carlo (MCMC), which is a future scope of the study. It should also be noted that our uncertainty quantification does not account for the uncertainty arising due to interpolation.

Although the numerical technique of gap-filling presented in the manuscript helps to achieve a reliable long-term ocean color gap-free dataset, this signals us towards the real underlying issue of the scarcity of the available in-situ observations, particularly in the Indian Ocean. If available consistently, these in-situ records of chlorophyll can be used to fill the gaps in satellite data. But the current distribution of in-situ measurements is not enough to fill the gaps observed in satellite data. Moreover, the scarcity of in-situ bio-Argos in the Indian Ocean limits us to further validate the satellite datasets. This demands immediate attention as the way ahead is to provide ecological forecasting for the Indian Ocean rim population which has a strong dependence on fisheries for their livelihood. We are hopeful that with the recent initiative of the Indian Ocean Observing System (IndOOS) program^72^ aimed to enhance the observations in the surface and subsurface tropical Indian Ocean by implementing the observing networks such as Argo floats, RAMA moorings, satellites, drifters^71^; accurate measurements will be gathered in the Indian Ocean. While this is one big collaborative step taken forward, more international participation is needed for the sustenance of the observational networks.

The importance of gap-free observations of biological variables before any actual data analysis could be carried out and has been already emphasized in the existing literature. Also, the uncertainty associated with analyzing the gap-filled datasets needs to be quantified for getting robust results. Our gap-filling of satellite ocean color is an attempt to make the long-term high-quality data more usable by computing parameters with uncertainty, quantitatively. Moreover, analyzing such datasets can lead to extracting timely information of the phenology of the ocean ecosystem, ocean–cyclone interactions and other biophysical interactions of a higher temporal frequency; thus proving beneficial for ocean model applications of ecological forecasting—presently a limitation in many of the earth system models. We further aim to use this dataset for the detection and attribution of phytoplankton phenology to anthropogenic climate change in the Indian Ocean.

## Supplementary Information

Supplementary Information.

## Data Availability

Daily synoptic fields of remotely-sensed chlorophyll concentrations (in mg/m^3^) at a 4-km spatial resolution are obtained from the European Space Agency Ocean Color-Climate Change Initiative (OC-CCI) version 4.2 (https://climate.esa.int/en/projects/ocean-colour/). Daily OLR fields are obtained from NOAA at one-degree resolution (https://psl.noaa.gov/). Levy’s dataset used in the analysis is available at http://www.nio.org. The in-situ Teledyne/Webb APEX—Argo floats deployed in the Arabian Sea are used from Ravichandran et al.^69^. The gap-free climatology of chlorophyll for the Indian Ocean generated in the current study is made available in GitHub repository (https://github.com/aditimodi/Gap_Free_Ocean_Color.git). And the 8-day composites of gap-free chlorophyll for the period 1998–2019 for the Indian Ocean are available from the corresponding author upon request.
