# Supplementary material for: Gap-filling of ocean color over the tropical Indian Ocean using Monte-Carlo method
Source: Sci Rep. 2022 Nov 1;12:18395. doi: 10.1038/s41598-022-22087-2 (PMC9626647; doi:10.1038/s41598-022-22087-2)
Supplement: Supplementary file 1 — Supplementary Information. [file 41598_2022_22087_MOESM1_ESM.pdf]

Gap-filling of ocean color over the tropical Indian Ocean using Monte-Carlo method

Aditi Modi*[1,2], Roxy M K[1], Subimal Ghosh[2,3]

Affiliations

1* *Centre for Climate Change Research, Indian Institute of Tropical Meteorology, Pune, India.* aditi.modi@tropmet.res.in

2 *IDP in Climate Studies, Indian Institute of Technology Bombay, India*

3 *Department of Civil Engineering, Indian Institute of Technology Bombay, India*

Contents of this file

Figures S1-S5

Table S1

Supplementary Figures

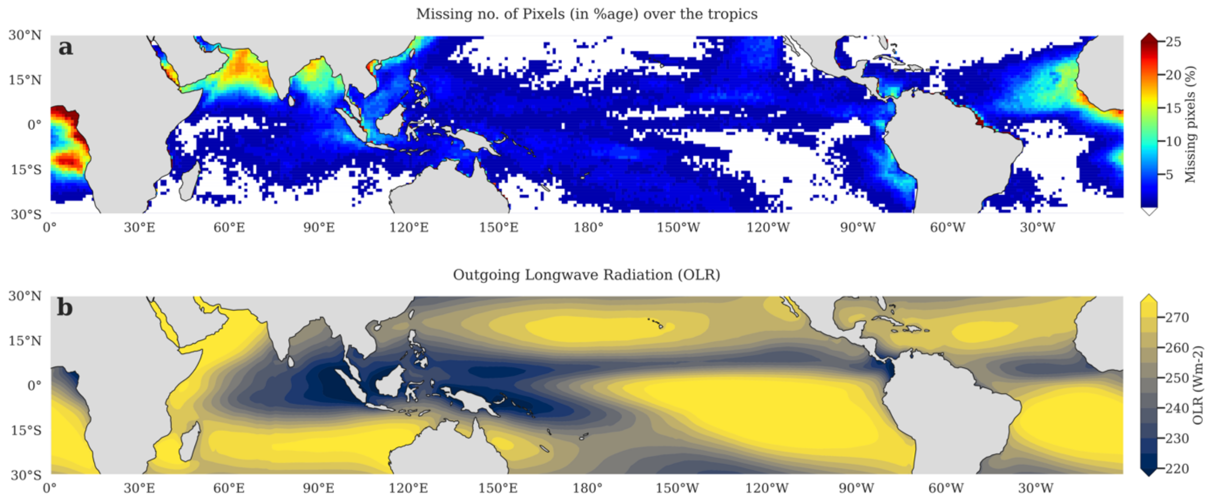

Supplementary Figure S1: (a) Number of missing pixels (in percentage) in 8-day composites of chlorophyll data from 1998-2019 over the tropical oceans. Gap-free pixels are marked in white. The pixels having more than 25% of missing values are represented in black; and (b) Climatological map of Outgoing Longwave Radiation (OLR, in W/m^2) in the tropical oceans for the period 1998-2019. The regions in dark blue are associated with weaker convection and those represented in yellow represent strong convection.

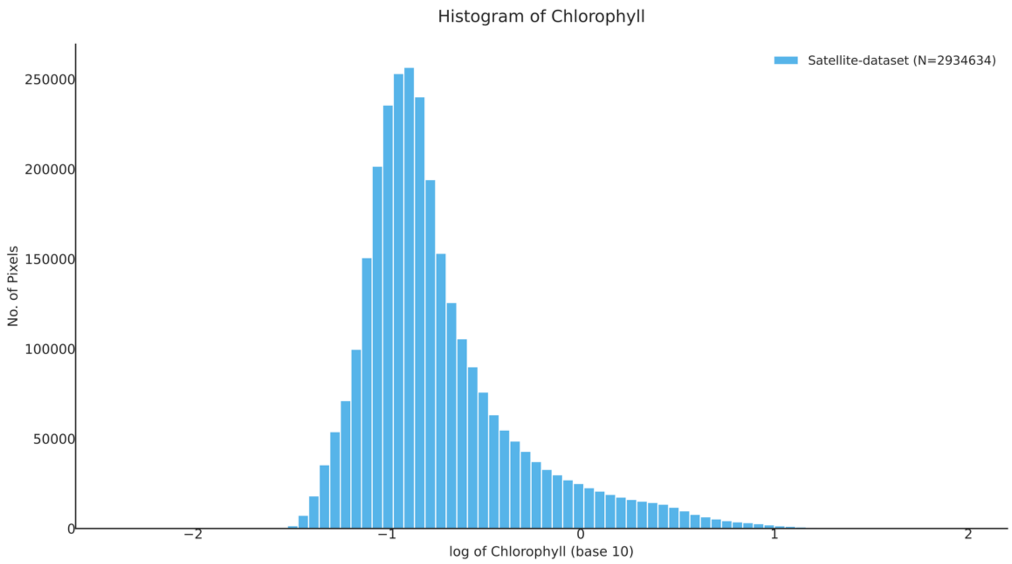

Supplementary Figure S2: Histogram of the logarithmic (base 10) of ESA v4.2 satellite chlorophyll data for the tropical Indian Ocean [40-120°E, 30°S-30°N]. The skewness of the log(chlorophyll) is 1.51.

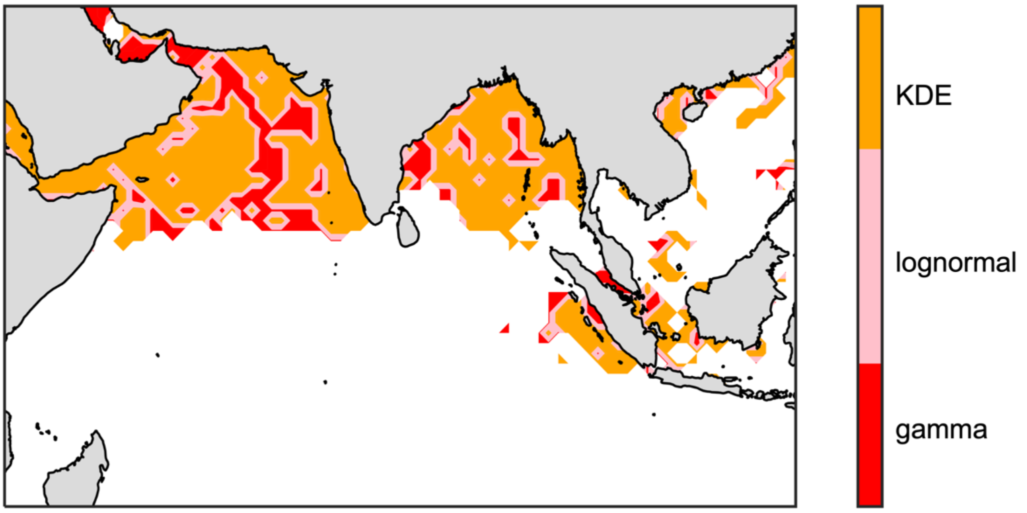

Supplementary Figure S3: The PDF identified at each grid. Each grid has been tested for both the parametric and non-parametric distributions. Most of the grids in the north Indian Ocean are fitted using a KDE (orange). Very few grids follow a lognormal (pink) and gamma (red) fir.

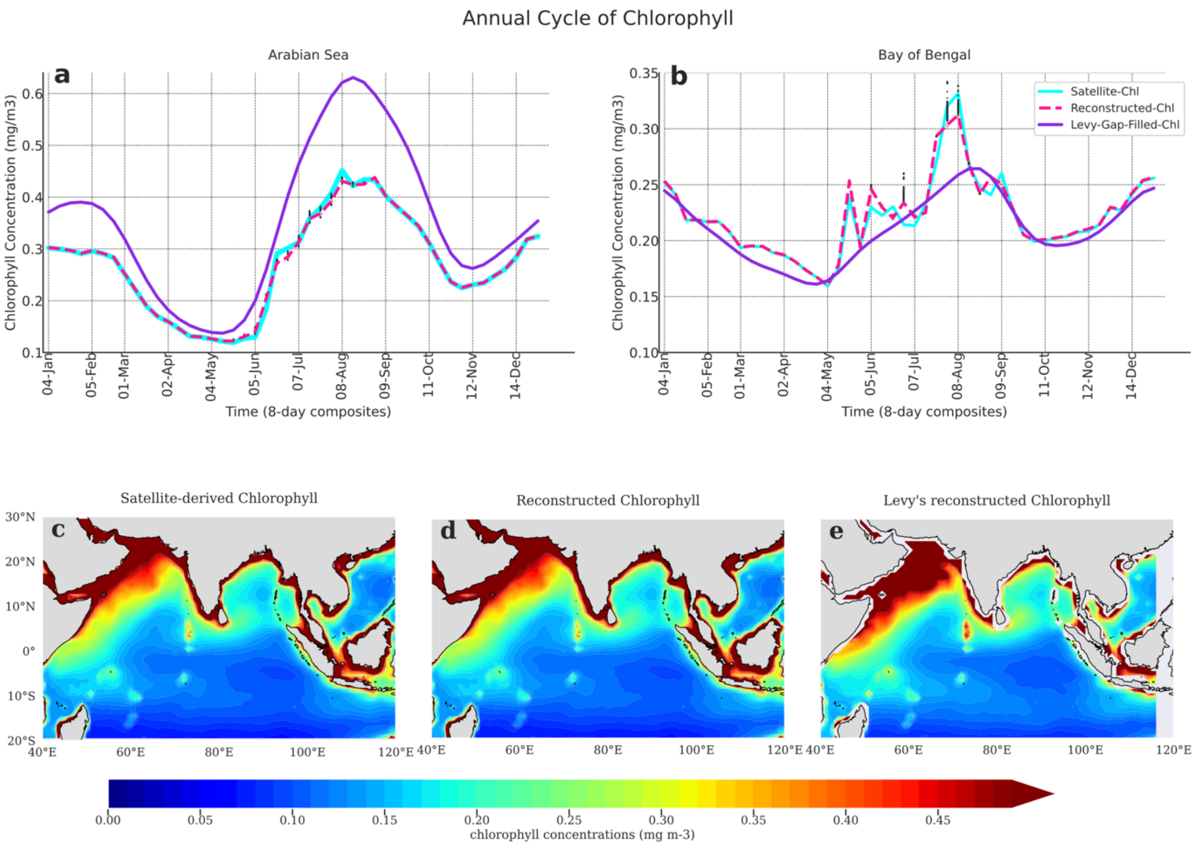

Supplementary Figure S4: Climatological annual cycle of reconstructed chlorophyll (8-day composites) for the period 1998-2005 in the (a) Arabian Sea [60°E-70°E,8°N-16°N], and (b) Bay of Bengal [85°E-95°E,8°N-16°N]. Light Blue line indicates ESA v4.2 satellite chlorophyll (original data); pink line represents the mean of gap-filled chlorophyll datasets (reconstructed data); and violet line represents the climatology of the gap-filled annual cycle reconstructed by Levy. The boxplot overlaid on the time series represents the range of values between the 25th and the 75th percentile. The black dots represents the outliers. Spatial distribution of chlorophyll concentration (in mg/m^3) in the tropical Indian Ocean for the period 1998-2005 in (c) satellite chlorophyll, (d) reconstructed chlorophyll using our methodology, and (e) Levy's reconstructed dataset.

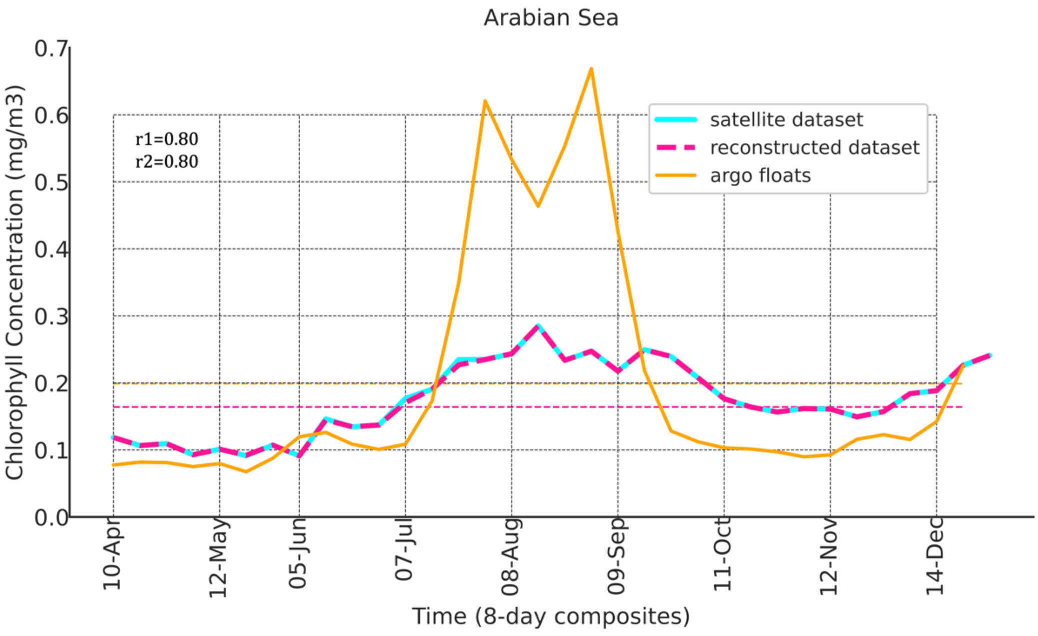

Supplementary Figure S5: Temporal evolution of chlorophyll-a (mg m^{-3}) during the year 2010, derived from an Argo float at 6 m depth (orange), reconstructed data (pink) and satellite data (cyan) for a region where the data points coincide in the Arabian Sea (66-68°E, 8-12°N). The horizontal dashed lines represents the mean of the datasets for the period April-December 2010. r1 represents the correlation coefficient value between the satellite dataset and Argo, and

r2 represents the correlation between the reconstructed dataset and Argo data. All the correlation values are significant at 95% confidence level.

Supplementary Table 1: Root mean Square Error (RMSE) values computed for interpolation in each dimension of longitude, latitude and time.

**Dimension used for interpolation**	**RMSE value**
Longitude	0.080
Latitude	0.086
Time	0.094
